# An Improved Electrochemical Aptasensor for Chloramphenicol Detection Based on Aptamer Incorporated Gelatine

**DOI:** 10.3390/s150407605

**Published:** 2015-03-27

**Authors:** Ezat Hamidi-Asl, Freddy Dardenne, Ronny Blust, Karolien De Wael

**Affiliations:** 1 AXES Research Group, Department of Chemistry, University of Antwerp, Groenenborgerlaan 171, B-2020 Antwerp, Belgium; E-Mail: Ezat.Hamidi-Asl@uantwerpen.be; 2 Sphere Research Group, Department of Biology, University of Antwerp, Groenenborgerlaan 171, B-2020 Antwerp, Belgium; E-Mails: freddy.dardenne@uantwerpen.be (F.D.); ronny.blust@uantwerpen.be (R.B.)

**Keywords:** aptamers, gelatine, chloramphenicol, electrochemical sensor

## Abstract

Because of the biocompatible properties of gelatine and the good affinity of aptamers for their targets, the combination of aptamer and gelatine type B is reported as promising for the development of biosensing devices. Here, an aptamer for chloramphenicol (CAP) is mixed with different types of gelatine and dropped on the surface of disposable gold screen printed electrodes. The signal of the CAP reduction is investigated using differential pulse voltammetry. The diagnostic performance of the sensor is described and a detection limit of 1.83 × 10^−10^ M is found. The selectivity and the stability of the aptasensor are studied and compared to those of other CAP sensors described in literature.

## 1. Introduction

In recent years, providing a stable immobilization of biomolecules while retaining their bioactivity has been a challenge in the preparation of biosensors [1,2]. Among different materials, hydrogels, have been one of the choices in this field and have shown some advantages in the context of the work of interest to our research group, *i.e.*, entrapping biomolecules in a protective environment aiming at sensing applications.

Hydrogels are three-dimensional networks of hydrophilic polymers that are able to swell incorporating large amounts of water and can be made to resemble the physical characteristics of soft tissues [3,4,5]. Gelatine is an example of a physically cross-linked hydrogel which is generally separated by the partial hydrolysis of collagen. Collagen is a group of naturally occurring proteins found in animals, especially in the fleshy tissues of vertebrates [6]. Gelatine derived from an acid-treated process is known as Type A or GelA, and gelatine derived from an alkali-treated process is known as Type B or GelB [7]. A variety of valuable studies focused on applications of the different kinds of gelatine have been published in recent years. Among them, Young *et al.* have published a review about gelatine and its roll in drug delivery [8]. Wang *et al.* have demonstrated formation of injectable and biodegradable colloidal gels upon mixing oppositely charged GelA and GelB nanospheres [9]. Lian *et al.* reported a novel injectable hydrogel as stem cell scaffold [10].

In this study, gelatine was addressed as a biocompatible matrix for an aptamer of chloramphenicol in the context of biosensor development. The electrochemical properties of gelatine and its applications have been studied by our research group [11,12,13,14,15,16]. Regarding the interesting properties of gelatine and unique characters of aptasensors, we have incorporated the aptamer into GelB gelatin both types are mentioned later to produce more sensitive and stable electrochemical biosensors.

Chloramphenicol (CAP) is a broad-spectrum antibiotic that is widely used in animals for the treatment of several infectious diseases because of its excellent antibacterial effects [17]. During the last decade several studies have been done for its detection and measurement [18]. For example Yan *et al*. designed and fabricated an aptasensor for detection of CAP in honey based on target-induced strand release [19]. Yadav *et al.* introduced another electrochemical aptasensor for CAP detection where the aptamer was immobilized onto poly-(4-amino-3-hydroxynapthalene sulfonic acid)-modified pyrolytic graphite [20].

In present work, a recently designed ssDNA aptamer has been used for CAP detection [21,22] and the electrochemical behavior of this sensing device towards the target molecule has been investigated. The data are compared with the results obtained from a CAP aptasensor without the protective gelatine matrix that were already published by our research group [23]. CAP has been chosen as a model target molecule because of its great importance in medicine and veterinary science [24,25]. Hereby, a new label-free aptasensor based on gelatine as immobilization matrix for measuring low levels of anti-infective agents was described.

## 2. Experimental Section

### 2.1. Apparatus

Electrochemical measurements were recorded by an Autolab potentiostat controlled by the NOVA 1.10 software package (Metrohm, Utrecht, The Netherlands). Morphological investigation of the electrode surface was done on a Fei Quanta 250 FEG Scanning Electron Microscope (Hillsboro, OR, USA). The SPEs was purchased from Metrohm and made of a gold working electrode (ϕ: 2 mm), a carbon counter electrode and a silver pseudoreference electrode.

### 2.2. Reagents

Chloramphenicol (Mw = 323.13 g·mol^−1^) was purchased from Sigma (Diegem, Belgium). Tris buffer containing 100 × 10^−3^ mol·L^−1^ NaCl, 20 × 10^−3^ mol·L^−1^ Tris HCl, 2 × 10^−3^ mol·L^−1^ MgCl_2_, 5 × 10^−3^ mol·L^−1^ KCl, and 1 × 10^−3^ mol·L^−1^ CaCl_2_ (pH = 7.6) was obtained from VWR (Leuven, Belgium) and used as a binding buffer solution. CAP binding aptamer sequence (5'-SH-(CH2)6-AGC-AGC-ACA-GAG-GTC-AGA-TGA-CTG-AGG-GCA-CGG-ACA-GGA-GGG-CAT-GGA-GAG-ATG-GCG-3') selected and designed at the SPHERE laboratory of the University of Antwerp [21,22] was purchased from Eurogentec (Seraing, Belgium). Type B gelatine (GelB, IEP = 5, Bloom strength = 257) isolated from bovine skin by the alkaline process and Type A gelatin (GelA, IEP = 8.8, Bloom strength = 202) isolated from porcine skin by the acid treatment were provided by Tessenderlo Chemie (Brussels, Belgium).

### 2.3. Procedure

In first step of procedure, the electrochemical pretreatment and biomodification of SPE were done. Prior to immobilization of thiolated DNA aptamer, a multiple-pulse amperometric pretreatment on the gold surface is carried out in a stirred 0.5 mol·L^−1^ H_2_SO_4_, 10 mmol·L^−1^ KCl solution with following triple-potential pulse sequence: −0.3 V for 3.0 s; 0.0 V for 3.0 s and +1.0 V for 1.5 s (15 cycles) [26,27]. The gold working electrode surface of SPE was then exposed to the mixture of aptamer (5 µM) containing GelB solution (5 w/v%; solved in Tris buffer at 40 °C). The percentage of the incorporation was 70:30 v/v% for aptamer:Gel solution in Tris buffer (pH 7.6). Their interaction was allowed to proceed for around 4 h, while the electrodes were stored in a wet chamber for protection from evaporation. Four hours was the best time for the immobilization of aptamer; because the minimum time for formation of a self-assembled monolayer of thiolated groups on the gold surface was 3 h [28,29,30] and the drop of gelatin was completely liquid in the wet chamber (≈40 °C) during this period. After that, the wet chamber was switched off and cooled down gradually to reach room temperature. At room temperature, the drop of apt/GelB became rigid.

The immobilization step was followed by dropping CAP solution (100 µL) on the surface of modified-gold SPE for 25 min. Prior to electrochemical measurements, the electrode surface was gently washed with 100 µL of Tris buffer. Then, the differential pulse voltammetry was performed in Tris buffer solution (pH 7.6). Because of gelatine layer is not stable enough after measurement and washing step, each modified SPE was used once. However, each measurement was repeated at least three times for each concentration.

## 3. Results and Discussion

**Scheme 1:**
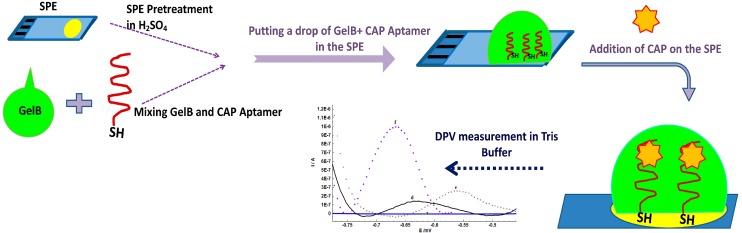
The preparation procedure of aptamer/GelB modified SPE for CAP detection.

Scheme 1 shows the electrochemical aptasensor based on incorporation of gelatine and aptamer for detection of CAP. In the absence of CAP, thiolated aptamers encapsulated in gelatine are partially unfolded, but linked to the gold surface by Au-S bonding. When CAP is introduced on the modified SPE, the aptamer switches its structure to bind CAP and bring the analyte molecules close to the electrode surface resulting in an enhanced electron transfer [31].

**Figure 1:**
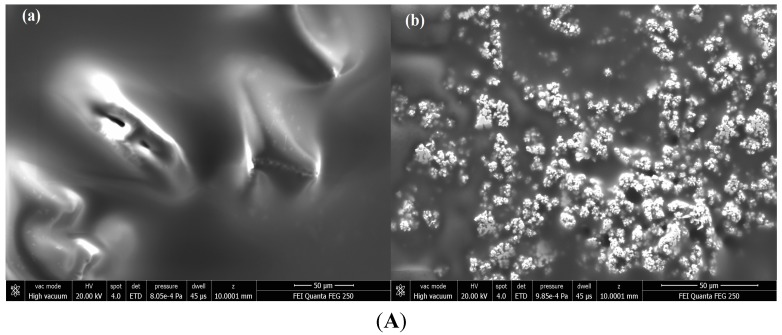

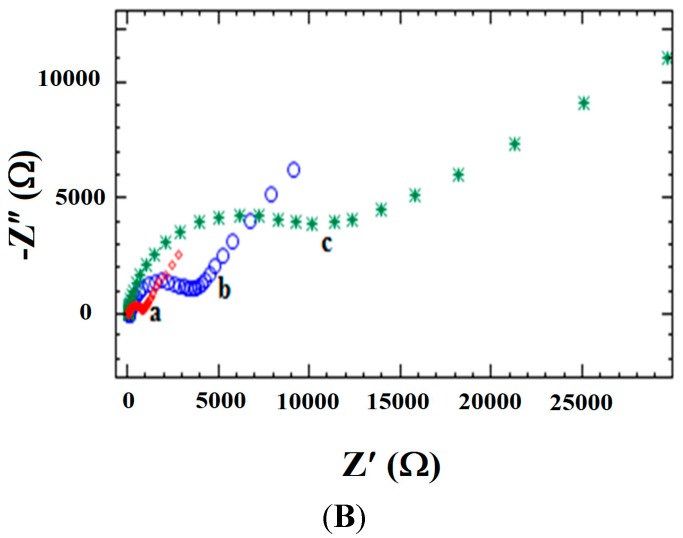
(**A**) SEM images of the working surface area after GelB immobilization without aptamers (a), and after aptamer incorporation (b); (**B**) The electrochemical impedance spectra of bare SPE (a), aptamer modified SPE (b) and aptamer/GelB modified SPE (c) in 10^−2^ mol·L^−1^ [Fe(CN)_6_]^4−/3−^ and bios potential 0.2 V.

To characterize the surface of the modified SPE, scanning electron microscopy (SEM) and electrochemical impedance spectroscopy (EIS) were performed. The morphology of the gelatine assembled gold electrode indicates a regular and porous structure (Figure 1A(a)). The gelatine film is compact, smooth and homogenous without grainy and porous structure, showing that an ordered matrix was formed. However, a rough, sponge like and irregular surface appears when aptamers have been added to the matrix (Figure 1A(b)), so a clear morphological change can be observed [32].

The EIS measurements were performed in the presence of [Fe(CN)_6_]^3−/4−^ couple as electroactive marker ions (Figure 1B). EIS measured data show that the electron transfer resistance increases in the following order: Bare SPE (Curve a), aptamer modified SPE (Curve b) and aptamer/GelB modified SPE (Curve c). This is attributed to the fact that ssDNA with negative charges on its phosphate backbone produces an electrostatic repulsive force on [Fe(CN)_6_]^3−/4−^ anions and prevents electrons from reaching the electrode surface.

The increase of electron transfer resistance indicates that the aptamers are successfully immobilized on the electrode surface. In the case of aptamer/GelB SPE, an additional barrier of negatively charged gelatine increases the electron transfer resistance which results in a larger semi-circle [33]. This evidence demonstrates the successful immobilization of the aptamer on the electrode surface and the blocking effect of the gelatine layer against unspecific redox active molecules.

**Figure 2:**
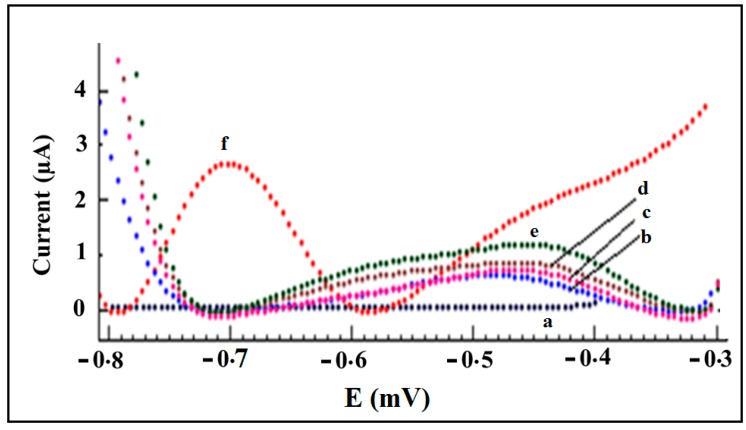
Differential pulse voltammograms of accumulated CAP at the surface of (a) bare gold SPE; (b) GelA modified SPE; (c) GelB modified SPE; (d) aptamer modified SPE; (e) aptamer/GelA modified SPE; (f) aptamer/GelB modified SPE in 20 mmol·L^−1^ Tris–HCl buffer solution (pH 7.6), pulse height: 0.05 V, scan rate: 20 mV·s^−1^. CAP concentration in accumulation step: 10^−8^ mol·L^−1^, time of accumulation: 25 min.

In order to investigate the role of the gel matrix in the efficiency of the aptasensor, differential pulse voltammetry (DPV) was selected as sensing technique. Figure 2 displays the differential pulse voltammograms of accumulated CAP (10^−8^ M) at the surface of bare gold SPE (Curve a: 0.00 µA), GelA-modified SPE (Curve b: 0.67 µA ± 0.02), GelB-modified SPE (Curve c: 0.801 ± 0.015 µA), aptamer-modified SPE (Curve d: 1.22 ± 0.06 µA), aptamer/GelA-modified SPE (Curve e: 0.86 ± 0.04 µA) and aptamer/GelB-modified SPE (Curve f: 2.71 ± 0.09 µA) in Tris buffer solution. The indicated currents reflect the peak current obtained in the voltammogram and can be explained as the irreversible reduction of the nitro group (NO_2_) present in CAP molecules, with formation of hydroxylamine (NHOH). As can be seen, there is no signal for this low concentration of CAP at a bare gold electrode (Curve a). After immobilization of gel at SPE, the DPV signal appears for accumulated CAP (Curves b and c). However, GelB-modified SPE shows a higher current signal than GelA. Following the modification of SPE by aptamer, the current of the DPV signal increased (Curve d). The combination of aptamer and GelA had no positive effect on sensor’s efficiency (Curve e), while the incorporation of aptamer and GelB shows a very good response toward the target molecules (Curve f) at the potential (*i.e.*, −0.7 V) at which the reduction of the nitro group in the CAP molecule structure is expected. This confirms the ability of the aptamer in its protective gelatine environment to capture the target molecules. Also, there is a pre-oxidation wave around −0.45 V that shows the intermediates in the redox reactions of chloramphenicol [34].

The increase in the DPV signal height after mixing the aptamer and GelB is most probably due to an increase in the charge transfer kinetics resulting in better reactivity of the aptamer in the biocompatible membrane towards the target. Another reason for GelB being more biocompatible could be the fact that GelB is more purified or contains less contaminants than GelA. Due to the incorporation of the aptamer in the GelB matrix, most of the aptamer sites will remain active during the immobilization and detection step and the mixture of aptamer/GelB shows a higher DPV signal than aptamer/GelA.

Considering Figure 2, the DP voltammogram of the aptamer/GelB modified SPE (Curve f) is almost three times higher than that of the aptamer-modified SPE (Curve d). Because of physical interactions between the aptamer chains and gelatine such as van der Waals forces and hydrogen bonds between amino acids, GelB is a good example of a physically cross-linked hydrogel. Therefore, the hydrophilic groups or domains which are hydrated make GelB a suitable matrix for the entrapment of the aptamers, while the incorporated aptamers are hydrated and can maintain their native configuration [35]. On the other hand, gelatine is generally categorized into mesh sizes 5 to 100 mesh [36] and a bloom strength of around 250 suggests an approximately 30 over 40 mesh size [37]. It means that the size of openings in the gelatine are around 590 µm [38]. Then, the size of holes is much bigger than the analyte and aptamer. Therefore, the analyte transfer to the surface of the electrode is not stopped by gelatine and it acts just as a cover matrix for the aptamer. However, to some extent diffusional hindrance can occur in the presence of gelatin in comparison to pure solution. But at the end, the positive effect of the gelatine cover is more than any negative effect of its hindrance.

Another reason for the increase of the reduction signal of accumulated CAP at the surface of aptamer/GelB modified SPE could be connected to the Pka of CAP. The isoelectric point for GelB is 5 and pKa value for CAP is 11.03 [39]. GelB has a negative charge and CAP has a positive charge in Tris buffer solution pH 7.0, respectively. Therefore, there is an electrostatic attraction between them and this is helpful for more accumulation of CAP molecules on the surface of the working electrode.

**Figure 3:**
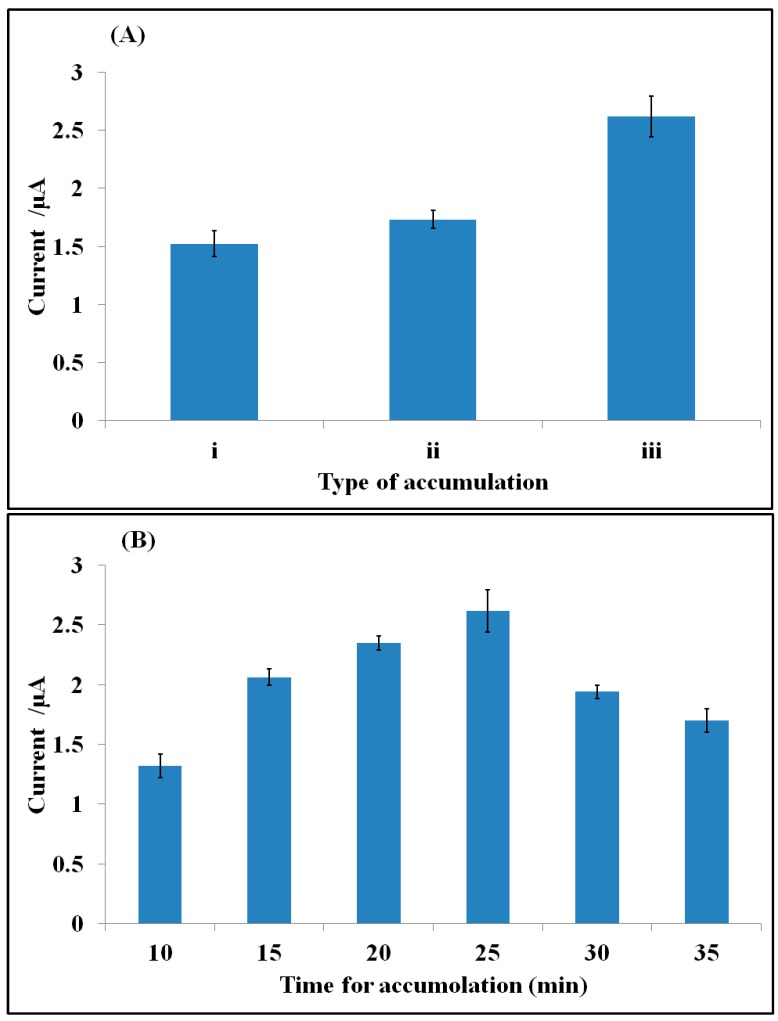
(**A**) Optimization the type of accumulation: i (stirring 1000 rpm), ii (stirring 100 rpm) and iii (wet drop); (**B**) the time for accumulation step in base of DPV’s height of accumulated CAP (10^−8^ M) at the surface of aptamer/GelB modified SPE in 20 mmol·L^−1^ Tris–HCl buffer solution (pH 7.6), pulse height: 0.05 V, scan rate: 20 mV·s^−1^.

To obtain the most sensitive detection, some parameters such as type and time of accumulation step were optimized for the electrochemical sensing of CAP. Figure 3 shows the height of the DPV signal of accumulated CAP (10^−8^ M) at the surface of aptamer/GelB modified SPE in 20 mmol·L^−1^ Tris–HCl buffer solution (pH 7.6) under different conditions. Three approaches were investigated to accumulate CAP on the surface of the aptamer/GelB electrodes. Firstly, the modified SPE was immersed in the CAP solution while the solution was stirred very fast (≈1000 rpm, i). Secondly, the CAP solution was stirred slowly (≈100 rpm, ii). The third one, 100 μL of the CAP solution was dropped on the modified SPE (wet drop, iii). The latter was proven to be the best to accumulate CAP because of the better opportunity for interaction between CAP, aptamer, GelB and the electrode surface (Figure 3A). Our assumption is the situation of stirring is not so convenient for accumulation of CAP molecules because for stirring, the SPE should be kept vertically in the solution; while, in the wet drop, the SPE is placed horizontally and 100 μL of solution is added at the surface of working electrode. It seems in this case, the CAP molecules have more chance to interact with aptamer. Also, the molecules of aptamer have more opportunity to change their configurations towards target molecules.

Also, the time of accumulation for CAP on the surface of the aptamer/GelB modified SPE was investigated during 10, 15, 20, 25, 30 and 35 min. When the accumulation time increased from 10 to 25 min, the signal of the accumulated CAP was enhanced. After that, no obvious changes in the DPV reduction current of CAP were observed. During 25 min, the gelatine layer completely absorbs water and swells as much as possible. After this period, some cracks start to appear at the surface of spherical gelatin drop and the gelatine layer broke up and somewhat lost its capability (Figure 3B). Therefore, 25 min was selected as the optimized time.

These cracks are completely obvious and can be seen by the naked eyes. The reason for the breakage is the “gelatin bloom”. Gel strength is traditionally referred as bloom. It is the force, expressed in grams, necessary to depress by 4 mm the surface of a gelatine gel with a standard plunger. Bloom is linked to the mechanical elasticity of the gel and is used to classify gelatine types. It generally ranges from 50 to 300 Bloom (Here, GelB = 257). Therefore, there is a limitation for each kind of gelatin to swell.

**Figure 4:**
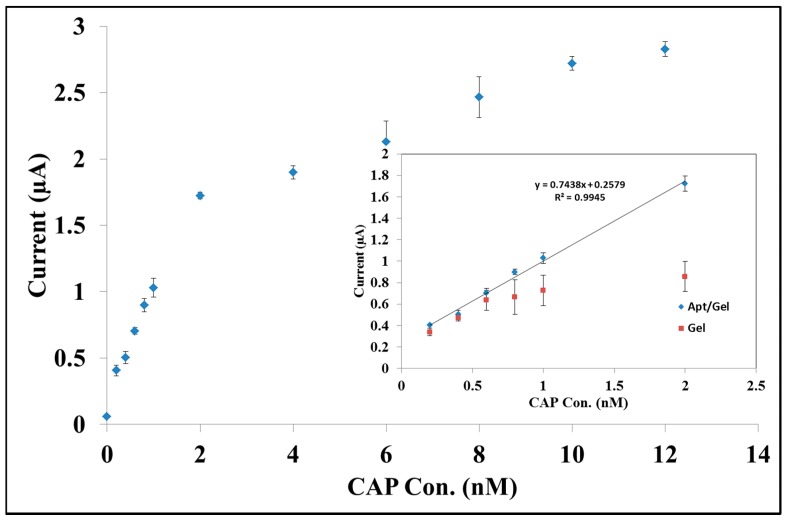
Variation of DPV signals *vs.* accumulated CAP solution (0.1 to 12 nmol·L^−1^) at the surface of aptamer/GelB modified SPE in 20 mmol·L^−1^ Tris–HCl buffer solution (pH 7.6), pulse height: 0.05 V, scan rate: 20 mV·s^−1^, time of accumulation: 25 min; inset*:* calculation of detection limit in base of linear dynamic range in 0.3 to 2.0 nmol·L^−1^ of CAP concentration; 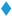 Apt/GelB and 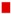 GelB modified SPE.

Figure 4 depicts the diagnostic performance of the aptasensor. To study the role of gelB as matrix in function of the biosensor, the CAP reduction signal was investigated on the aptamer/GelB-modified SPE. It can be seen that the peak current increases with increasing CAP concentration (0.10 to 12.0 nmol·L^−1^) with two different slopes [40,41,42]. The peak current shows a linear relationship with the concentration of CAP in the range from 0.30 to 2.0 nmol·L^−1^ (inset). The calculated detection limit is 1.83 × 10^−10^ mol·L^−1^ based on three times standard deviation of the blank divided by the slope of the calibration curve (3σ/m).

When these experiments were done before in our lab without using gelatine the calculated LOD was 1.60 × 10^−9^ mol·L^−1^ [23]. This means the sensitivity of the aptasensor is increased because gelatine can keep the analyte molecules closer to each other and closer to the surface area of the working electrode. As seen in the inset, a calibration plot obtained at the surface of gelatin- modified SPE (without aptamer) shows no sensitivity to the target in comparison with the Apt/GelB-modified electrode.

To measure the net aptamer affinity that is responsible for CAP detection and distinguish the electrostatic interaction of gel from the aptamer affinity, ΔI = (I_1_ − I_2_) is calculated as below:

I_1_: DPV signal of accumulated CAP solution at the surface of aptamer/GelB-modified SPE.

I_2_: DPV signal of accumulated CAP solution at the surface of GelB-modified SPE.

**Figure 5:**
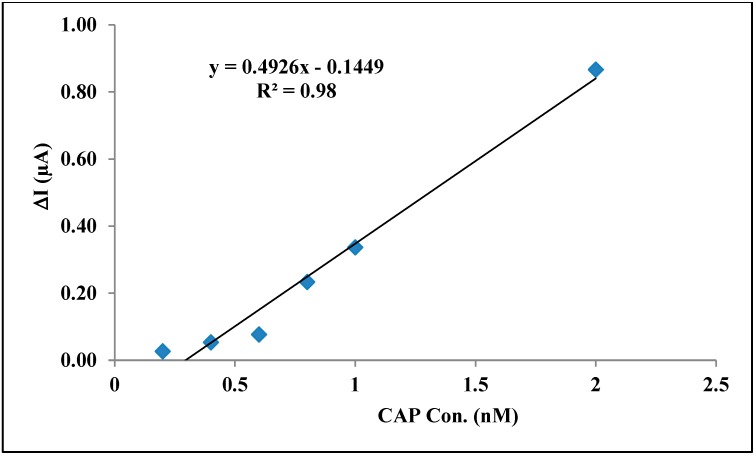
ΔI (µA) *vs.* accumulated CAP solution (0.2 to 2 nmol·L^−1^) at the surface of modified SPE in 20 mmol·L^−1^ Tris–HCl buffer solution (pH 7.6).

Figure 5 shows the variation of ΔI (µA) [the difference between currents] *vs.* accumulated CAP solution (0.2 to 2 nmol·L^−1^). As seen in this figure, a good linear relation between current and CAP concentration with a correlation coefficient of 0.98 that is acceptable for analytical goals is still there.

The assay of the target in spiked samples was investigated by detecting CAP in skimmed cow’s milk samples. The CAP reduction peak occurred in the expected potential range with the aptamer/GelB-modified SPE compared to the reduction current obtained at an aptamer immobilized electrode without gelatine as protective matrix, suggesting an enhanced sensitivity of the developed sensor. Recovery values list in Table 1 indicate the ability of the developed aptasensor for CAP detection in spiked samples. However, as seen in the third row of the table, the efficiency of this aptasensor was weak at lower concentrations based on the recovery and relative standard deviation R.S.D values.

**Table 1:** Analysis of CAP at different concentrations in spiked samples.

Sample	Added (M)	Detected (M)	Recovery (%)	R.S.D. (%)
1	1.0 × 10^−8^	9.30 ± (0.32) × 10^−9^	93	3.44
2	5 × 10^−9^	4.20 ± (0.20) × 10^−9^	84	4.76
3	1.0 × 10^−9^	8.10 ± (0.47) × 10^−10^	82	5.80

Table 2 shows the capability comparison of this sensor with others reported in the literature. As can be seen, our modified sensor displays comparable LOD for CAP detection because of the biocompatible interaction between the aptamer and gel. However, the examples in [20,43] of this table have approximately better LOD compared with the present work because of their modification.

The biosensor in [20] is a chemically modified sensor in base of polymerization of 4-amino-3-hydroxynaphthalene sulfonic acid. This polymeric layer is a conductive membrane that can increase the electron transfer and height of signals. This results in a better LOD. Also, a conducting polymeric layer combined with nanoparticles described in [43] causes better biosensor efficiency. However, the advantages of the sensor presented in this work are that it is easy-to-make, easy-to-use, and is stable in storage (after one month in a refrigerator, 90% of the initial current signal could be obtained).

**Table 2:** Comparison of the efficiency of some modified electrodes in the detection of CAP.

Number	Electrode	Modification	Detection Method	LOD	Ref.
1	Gold	Biotinylated detection probe	Square wave voltammetry (SWV), Impedance spectroscopy	0.29 × 10^−9^ mol·L^−1^	[19]
2	pyrolytic graphite	p-AHNSA	Cyclic voltammetry (CV), SWV	0.02 × 10^−9^ mol·L^−1^	[20]
3	Gold	Thiolated aptamers	CV, SWV	1.6 × 10^−9^ mol·L^−1^	[23]
4	Glassy carbon	Gold nanoparticles, dendrimers, and cadmium sulfide nanoparticles	CV	45 pg·mL^−1^ or 1.39 × 10^−10^ mol·L^−1^	[43]
5	Glassy carbon	ELISA (an enzyme label)	DPV	0.064 μg·L^−1^ or 1.98 × 10^−10^ mol·L^−1^	[44]
6	Carbon paste	Molecular imprinted polymer	Potentiometry	10^−6^ mol·L^−1^	[45]
7	Carbon paste	Molecular imprinted polymer and non-imprinted polymer	CV, DPV, SWV	2 × 10^−9^ mol·L^−1^	[46]
8	Gold Screen printed electrode	Thiolated aptamer + GelB	DPV	1.83 × 10^−10^ mol·L^−1^	Present work

Because disposable SPE were used for these experiments, each electrode could be used one time for each measurement. However, the reproducibility of results was good as long as we used the same type of SPE (from one company) for our experiments.

## 4. Conclusions

In conclusion, an aptamer has been incorporated into a gelatine matrix with the aim of developing a more stable and sensitive aptasensor for biosensing. The hydrophilic network of gelatine provides a suitable micro-environment for aptamer immobilization, which facilitates the electron exchange between the target molecules and the electrode. The gelatine films can be easily prepared and are stable over a long period in a refrigerator (storage stability). Aptamers can be effectively immobilized on a gold electrode surface by incorporation within the porous network of gelatine. The results showed that the three-dimensional and hydrated environment of gelatine helped increase the sensitivity of the developed sensor by holding the aptamer onto the electrode surface and preventing poisoning of the electrode surface. Sensors modified with gelatine and aptamers showed higher sensitivity toward CAP compared to the situation without gelatine as protective matrix. It is hoped that the encouraging results obtained by a mixture of aptamer and gelatine based on their intrinsic properties and the versatility of the procedure will generate new applications in diagnostics as well as food analysis. The combined use of aptamers with hydrogels is still a novel concept that has a huge potential for numerous clinical applications and drug delivery.
